# Why responses to immune checkpoint inhibitors are heterogeneous in head and neck cancers: Contributions from tumor-intrinsic and host-intrinsic factors

**DOI:** 10.3389/fonc.2022.995434

**Published:** 2022-10-18

**Authors:** Zhangguo Chen, Jessy John, Jing H. Wang

**Affiliations:** ^1^ UPMC Hillman Cancer Center, Division of Hematology and Oncology, Department of Medicine, University of Pittsburgh, Pittsburgh, PA, United States; ^2^ Department of Immunology, University of Pittsburgh, Pittsburgh, PA, United States

**Keywords:** immune checkpoint inhibitors, tumor heterogeneity, immunological heterogeneity, individualized anti-tumor immune responses, TCR repertoire

## Abstract

Immune checkpoint inhibitors (ICIs) have revolutionized cancer treatment including in head and neck squamous cell carcinomas (HNSCCs); however, only a fraction of HNSCC patients respond to ICI, whereas the majority fail to do so. The mechanisms underlying such variable responses remain incompletely understood. A better understanding of such mechanisms may broaden the spectrum of responding patients and enhance the rate of ICI response. HNSCCs exhibit a high level of genetic heterogeneity, manifested as mutations or amplifications of oncogenes (e.g., *PIK3CA*) and mutations of tumor suppressor genes (e.g., *TP53*). The immune tumor microenvironment (TME) of HNSCCs also varies significantly in composition and in relative abundance of distinct immune subsets such as CD8 tumor-infiltrating lymphocytes (TILs) or tumor-associated macrophages (TAMs), which represents a high degree of immunological heterogeneity. Here, we briefly discuss how heterogeneous ICI responses may be attributed to tumor-intrinsic factors, including genetic, transcriptional, and functional variations in tumor cells, and host-intrinsic factors, including cellular composition of the TME (e.g., CD8 TILs and TAMs), and host-intrinsic differences in the T cell receptor (TCR) repertoire of CD8 TILs. We also discuss the potential impact of these factors on designing strategies for personalized immunotherapy of HNSCCs.

## Introduction

Head and neck cancer (HNC) is a heterogeneous group of cancers arising from the mucosal surfaces of the upper aerodigestive tract including sinonasal and oral cavities, nasopharynx, oropharynx, hypopharynx, and larynx (1). Collectively, HNC is the sixth most prevalent cancer worldwide with 890,000 new cases and 450,000 deaths in 2018 (1, 2). In the United States (US), HNC accounts for 3-4% new cases of all cancer types (3), with 90% of cases being head and neck squamous cell carcinoma (HNSCC). The risk factors for HNSCC include carcinogens (e.g., tobacco and alcohol) and human papilloma virus (HPV) (1, 4). Hence, HNSCC can be classified as HPV^+^ or HPV^−^ based on the distinct etiological factors. In general, HPV^+^ HNSCC patients exhibited better overall survival (OS) than HPV^−^ patients, the latter showing worse prognosis (1, 4). During the past few decades, the incidence of HPV^+^ oropharyngeal HNSCCs has been increasing rapidly in the US (1, 4).

Extensive genomic and multi-omic studies performed using HNSCC patient samples conclude that HNSCCs displayed a high level of tumor heterogeneity including genetic, epigenetic, transcriptional, and functional variations between tumors or within tumors. A comprehensive multi-omic study revealed that HPV^−^ HNSCCs can be clustered into three major subtypes by integrating copy-number, RNA, miRNA, protein, and phosphor-peptide data. The three subtypes include high chromosome instability (CIN), Basal, and Immune (5). CIN cluster was associated with heavy smoking and exhibited the worst prognosis (5). On the other hand, Immune cluster was enriched with tumors where smoking evidence was weak and associated with higher immune scores (5). Another study also employed multi-omic approaches to compare different types of SCCs in lung, cervix and head and neck, and showed that HNSCCs appeared to scatter broadly instead of localizing to discrete TumorMap islands and distributed into distinct iClusters (6). These findings are consistent with other large genomic studies showing that HNSCCs harbor a high level of genetic and epigenetic alterations (7–9).

HNSCC datasets of the Cancer Genome Atlas (TCGA) identified many commonly occurring genetic alterations in both HPV^−^ and HPV^+^ HNSCCs. The most commonly mutated gene in HNSCC is tumor suppressor gene *TP53* (8), encoding a transcription factor regulating DNA repair, cell cycle, senescence, and apoptosis (10). Over 80% of HPV^−^ HNSCCs harbor *TP53* mutations; in contrast, *TP53* mutations almost never occur in HPV^+^ HNSCCs (~3%) (8), due to p53 protein degradation induced by the HPV E6 oncoprotein (11, 12). Another commonly mutated gene in HNSCCs is *PIK3CA*, encoding a catalytic subunit (p110α) of phosphoinositide 3-kinase (PI3K). *PIK3CA* genetic alterations, including both point mutations and gene amplification, affected both HPV^+^ and HPV^−^ HNSCCs (56% and 34%, respectively) (8, 13), making *PIK3CA* the most frequently mutated gene in HPV^+^ HNSCCs. We evaluated the TCGA HNSCC dataset and found that the patients with *PIK3CA* amplification and gain (*PIK3CA^Amp^*) had a much greater chance of harboring *TP53* mutations (14). Moreover, *PIK3CA* ^Amp^/*TP53* ^Mut^ group exhibited significantly worse survival compared to *PIK3CA* ^WT^/*TP53* ^WT^ groups (14).

Prior studies have generated murine models that mimicked the alterations of *PIK3CA* and/or *p53* in HNSCCs (15–17); however, none of the previous studies showed that genetic alterations in these two genes can spontaneously induce HNSCC development. We recently established a genetically engineered mouse model by deleting *p53* and constitutively activating *PIK3CA* in mouse keratin 15-expressing (K15^+^) stem cells, which leads to the spontaneous development of multi-lineage tumors including SCCs, termed keratin-15-p53-PIK3CA (KPPA) tumors (14). Furthermore, we derived transplantable daughter cell lines from KPPA tumors, which may provide a platform for testing new therapeutic strategies in HNSCCs (18).

HNSCCs also exhibited a high level of immunological heterogeneity, evidenced by a highly variable immune tumor microenvironment (TME) (19–21). Prior studies showed that the infiltration extent of CD8 tumor-infiltrating lymphocytes (TILs) correlated with HNSCC prognosis (22–25), while myeloid cell infiltration may contribute to worse survival and metastasis (19). We uploaded RNA-seq data of TCGA-HNSCC patients onto CIBERSORT and found that HNSCCs with *PIK3CA^Amp^*/*TP53^mut^* have significantly lower expression of gene signatures for CD8 T cells and activated natural killer (NK) cells but significantly higher expression of macrophage gene signature, compared with HNSCCs lacking both mutations (14). HNSCC is also characterized by defects in DNA repair pathways that can be induced by drug perturbation such as PARP inhibitor (26) or by loss of tumor suppressors such as *Smad4* (27). *SMAD4* loss has been associated with downregulation of FANC/BRCA genes in HNSCC harboring increased genomic instability (26). Genomic instability in HNSCC may generate cytosolic double-stranded DNA (dsDNA), which can be sensed by STING protein (28). STING activation subsequently induces type I interferon (IFN) and TNFα production, and triggers anti-tumor innate immunity (26). It would be of great interest to further elucidate whether a different level of genomic instability influences the level of CD8 TILs in HNSCC.

## Differential responses to immune checkpoint inhibitors (ICI) in human HNSCC patients

So far, two ICIs, namely, nivolumab and pembrolizumab, both of which are anti-PD-1 monoclonal antibodies (mAbs), were approved by FDA for treating recurrent/metastatic (R/M) HNSCCs (29–31). However, only a fraction of HNSCC patients (10-20%) responded to ICI while others failed to do so (29–31). KEYNOTE-048 trial tested ICI for treating R/M HNSCCs in first line therapy (32). The study reported positive results in OS, thus, ICI emerged as the new standard-of-care (SOC) therapy (32). According to the observed efficacy and safety, pembrolizumab (pembro) plus platinum and 5-fluorouracil (5-FU) serves as a proper first-line therapy for R/M HNSCC while pembro monotherapy is an appropriate first-line treatment for PD-L1^+^ R/M HNSCC (combined positive score (CPS)>1). However, many issues remain to be addressed. While pembro plus chemotherapy increased OS, it did not significantly increase overall response rate (ORR) between Pembro+Platinum+5-FU (35.6%) vs. EXTREME (cetuximab+Platinum+5-FU) (36.3%), moreover, it significantly reduced the duration of response (DOR) between Pembro only (22.6 months) vs. Pembro+Platinum+5-FU (6.7 months) (32). Hence, other strategies are worth exploring to improve ORR and extend DOR. With regard to pembro monotherapy, the ORR still remained low, and the progressive disease rate was 40.5% (32); thus, it is critical to enhance treatment efficacy and better stratify and identify patients who would benefit the most from ICI treatment. Besides chemotherapy, HNSCC patients are often treated by radiation therapy (RT), whose critical role has been investigated and reviewed extensively (33–38).

For treating locally advanced HNSCCs, a randomized, double-blind, and placebo-controlled phase 3 trial compared avelumab (anti-PD-L1) plus chemoradiotherapy (CRT) vs. CRT alone, where anti-PD-L1 was administrated concurrently with CRT; however, the trial reported negative results (39). Therefore, more effective, and novel combinatorial strategies are needed to better treat locally advanced HNSCCs. Nivolumab was employed to treat at-risk, previously untreated, resectable HPV^+^ and HPV^−^ HNSCC in a neoadjuvant setting (40–43). One of the neoadjuvant trials compared HPV^+^ vs. HPV^−^ HNSCCs and showed that HPV^+^ HNSCC patients responded better to neoadjuvant nivolumab, although the response rate was low in both HPV^+^ and HPV^−^ HNSCCs (40) compared to other cancer types (44, 45). Less responsiveness in HNSCC is consistent with its immunosuppressive TME (19, 46). Recent and ongoing ICI trials in HNSCCs were extensively reviewed (31, 47). However, it remains poorly understood why some patients responded to ICIs while others failed to do so (48–50). A better understanding of the mechanisms underlying such variable responses may broaden the spectrum of responding patients and enhance the rate of ICI response in HNSCCs.

It remains unresolved whether HPV^+^ HNSCC patients responded to ICI better than HPV^−^ ones because clinical studies reported inconsistent results (**Table 1**). In the KEYNOTE-012 trial with pembro as first-line or subsequent-line treatment, R/M HPV^+^ HNSCC patients showed a higher ORR than HPV^−^ patients (25% vs. 14%) (51). The expansion cohort of KEYNOTE-012 reported an even higher ORR (32% vs. 14%), and favorable 6-month progression-free survival rate (37% vs. 20%) and 6-month OS rate (70% vs. 56%) in HPV^+^ HNSCC patients compared to HPV^−^ ones (52). Similarly, a higher ORR was reported for HPV^+^ patients in CheckMate 141 trial (53) using nivolumab (HPV^+^ vs. HPV^−^: 17.2% vs. 14.3%) and in HAWK study (54) using durvalumab (HPV^+^ vs. HPV^−^: 29.4% vs. 10.8%). However, Keynote-055 trial using pembro showed a similar ORR in R/M HNSCC patients regardless of HPV status (HPV^+^ vs. HPV^−^: 16% vs. 15%) (55). A similar ORR was also reported for HPV^+^ and HPV^−^ HNSCC patients (HPV^+^ vs. HPV^−^, 15% vs. 17%) in NCT01375842 trial using Atezolizumab (56). Two recent meta-analysis integrated all the clinical data and showed that anti-PD-1/PD-L1 therapy favored a higher response rate in HPV^+^ than HPV^−^ HNSCC patients (57, 58). While these studies collectively suggest an increased sensitivity of HPV^+^ HNSCCs to ICI treatment, future clinical trials with a greater number of patients probably are needed to completely resolve this issue.

**Table 1:** Summary of clinical trial studies for differential ICI response in HPV+ vs HPV− HNSCC patients.

Study	Pub Year	Phase	Treatment	Total patient (N)	HPV status (n)	ORR	Median os (months)	Ref #
Keynote-012	2016	lb	Pembrolizumab (anti-PD-1)	60	HPV-pos (23) HPV-neg (37)	25% 14%	Not reached 8	51
Keynote-012, Expansion cohort	2016	lb	Pembrolizumab (anti-PD-1)	132	HPV-pos (28) HPV-neg (104)	32% 14%		52
CheckMate 141, 2-year update	2018	III	Nivolumab (anti-PD-1)	240	HPV-pos (64) HPV-neg (56)	17.2% 14.3%	9.1 7.7	53
HAWK Trial	2019	II	Durvalumab (anti-PD-L1)	112	HPV-pos (34) HPV-neg (65)	29.4% 10.8%	10.2 5	54
Keynote-055	2017	II	Pembrolizumab (anti-PD-1)	171	HPV-pos (37) HPV-neg (131)	16% 15%		55
NCT01375842	2018	la	Atezolizumab (anti-PD-L1)	32^a^	HPV-pos (13) HPV-neg (12)	15% 17%		56

^a^In NCT01375842, four patients with nasopharyngeal cancer were excluded from the HPV analysis population and three patients with unknown status.

## Differential responses to ICI in mouse HNSCC models

To better delineate why ICI treatment results into variable responses, we employed another syngeneic mouse model of SCC, namely, the A223 tumor with *Smad4* deletion, which has been characterized previously (59–61). While *Smad4* mutations do not occur commonly in HNSCCs, *Smad4* deletion is frequently observed in a large portion of HNSCC samples (62). Given Smad4 plays a key role in TGFβ signaling and Smad4-deficient SCCs elevated TGFβ level (63), we investigated whether Smad4^-/-^ SCCs responded to combined TGFβ/PD-L1 blockade differentially. We found that distinct immune TME profiles of therapeutic responders emerge in combined TGFβ/PD-L1 blockade-treated SCC (64). Responders contained more CD8 TILs and these CD8 TILs also exhibited more potent effector functions compared to non-responders (64). Additionally, responders harbored more M1 macrophages and less resident monocytes in the TME, compared to non-responders (64). The expression of major histocompatibility complex (MHC) was higher on responder myeloid cells or dendritic cells than non-responder counterparts (64). Nevertheless, it remains unclear why certain tumor recipients emerged as responders while others as non-responders.

To test whether oncogenic driver mutations affect differential ICI responses, we employed the two established KPPA tumor lines (TAb2 vs. TCh3), both of which harbor *TP53* deletion and *PIK3CA* hyperactivation. When transplanted into C57BL/6 recipients, TAb2 and TCh3 tumors responded to anti-PD-L1 differentially, with the former completely lacking response and the latter being relatively sensitive (18). We employed conventional flow cytometry and single-cell RNA-sequencing to identify the difference in TILs. We found that TAb2 and TCh3 KPPA tumors exhibited heterogeneous immune profiles pre-existing treatment that dictated their unresponsiveness or sensitivity to anti-PD-L1 (18). Others have established murine HNSCC cell lines from primary 4NQO-induced tumors in the tongue of C57BL/6 (B6) mice, which were designated 4MOSC, short for 4NQO-induced murine oral squamous cells (65). Some of the 4MOSC cell lines also exhibited variable responses to anti-PD-1 (65). Overall, ICI treatment can result in heterogeneous outcomes in preclinical models. These findings are consistent with clinical observations.

## Tumor-intrinsic factors influence differential ICI responses

**Figure 1:**
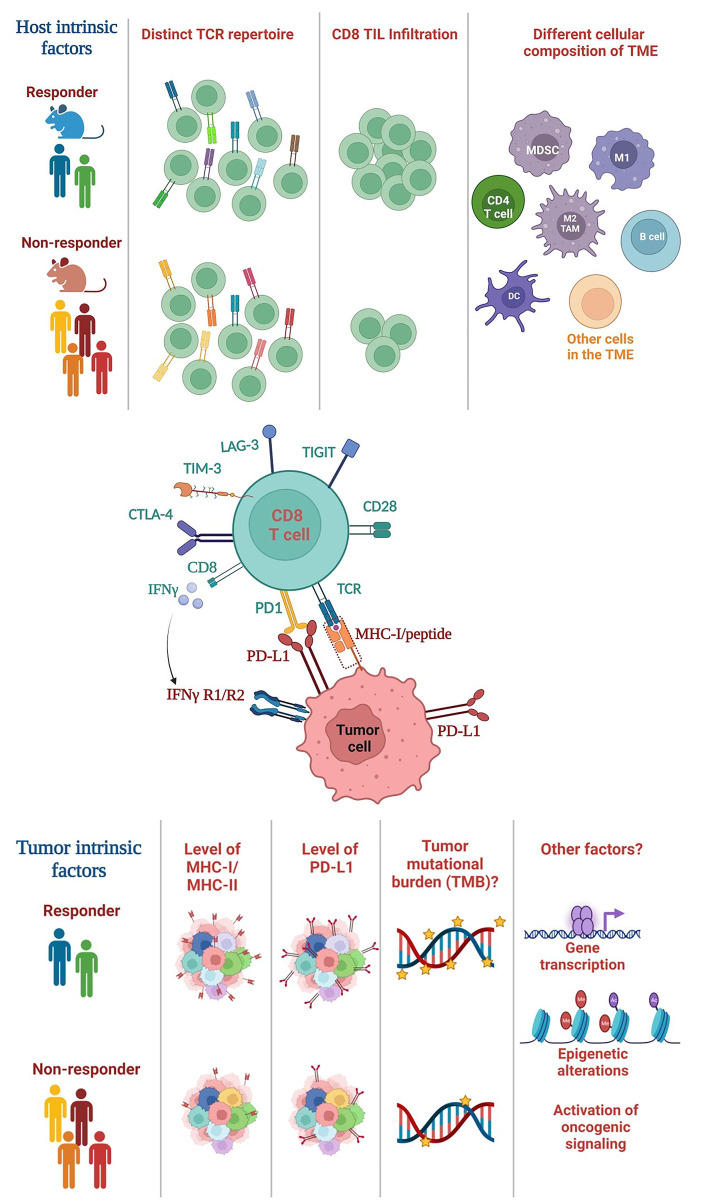
Summary of host-intrinsic and tumor-intrinsic factors that may influence the heterogeneous outcomes of ICI treatment. (Top) Host-intrinsic factors: (1) distinct TCR repertoires in responders vs. non-responders, (2) different level of CD8 TIL infiltration, (3) different cellular composition of the TME. Various subsets of immune cells are shown including CD4 T cells, B cells and myeloid cells, whereas many subsets of other cells in the TME are not shown including NK cells, Tregs, fibroblasts etc. TCR, T cell receptor; TIL, tumor-infiltrating lymphocyte; TME, tumor microenvironment. (Middle) The interaction between CD8 T cells and tumor cells. CD8 T cells recognize the MHC class I/peptide complex present on tumor cell surface. CD8 T cells can express various exhaustion markers including PD-1, TIM-3, LAG-3 etc. CD8 T cells can also secrete cytokines such as IFN-γ, while tumor cells express IFNγ receptor (IFNγR). (Bottom) Tumor-intrinsic factors: (1) differential level of MHC class I or class II expression, (2) PD-L1 expression on tumors (shown) or other cells in the TME (not shown), (3) the level of TMB, and (4) other potential factors such as genetic or epigenetic alterations in tumor cells, differential transcriptomes; activation of different oncogenic signaling pathways. TMB, tumor mutational burden.

Extensive prior studies have suggested a critical role of tumor-intrinsic factors in mediating differential responses to ICI, including tumor mutational burden (TMB), PD-L1 expression, or genetic and epigenetic differences in tumor cells themselves (**Figure 1**). For instance, TMB was shown to correlate to ICI efficacy in melanoma and non-small cell lung cancer (NSCLC) (66–68). However, the role of TMB in HNSCCs remains controversial. While studies showed that TMB^high^ HNSCCs responded to ICI treatment better (69–71), conflicting data showed that TMB did not correlate with ICI response (72, 73). The pathogenesis of HPV^−^ HNSCCs are strongly associated with carcinogens (e.g., tobacco); thus, HPV^−^ HNSCCs contain a high level of TMB yet they failed to respond to neoadjuvant nivolumab treatment as well as melanoma or NSCLCs (40). Overall, these data indicate that the TMB level does not fully explain differential ICI responses in HNSCCs. It remains unclear why the role of TMB in HNSCCs differ from melanoma and NSCLC. It is possible that HNSCC is a type of cancers that are inherently heterogeneous (5, 6). It is also possible that HNSCCs may have a lower level of CD8 TIL infiltration before ICI treatment compared to other cancers such as melanoma.

PD-L1 expression has been used as a biomarker for correlating ICI responses in HNSCCs. Clinical trial data showed that PD-L1 expression is predictive of the response rate and survival if the CPS was used based on expression of tumor and TME (31). However, the PD-L1 expression based on the tumor proportion score (TPS) does not predict ICI response rate or survival (31), suggesting that tumor-derived PD-L1 expression is less important than combined PD-L1 expression from both tumor and TME. Nevertheless, CPS cannot accurately predict ICI responses and additional accurate biomarkers are needed. Tumors may respond to ICI better if tumor cells are capable of increasing the PD-L1 expression in response to inflammatory stimuli abundant in the immune TME. In line with this idea, studies from melanoma suggest that conserved IFN-γ signaling drives clinical response to ICI treatment (74), although it remains unknown whether this mechanism also operates in HNSCCs. Our studies suggest that the ability of tumor cells to upregulate PD-L1 expression in response to IFN-γ stimulation may serve as a predictive marker for ICI responses (18), consistent with prior findings that IFN-γ and expanded immune gene signatures correlated with better ICI response in HNSCCs (75).

We uncovered tumor-intrinsic differences that may underlie the differential responses to ICI by employing two KPPA tumor lines, TAb2 vs. TCh3 (18). TAb2 tumors failed to respond to anti-PD-L1, whereas TCh3 tumors were relatively sensitive (18). Unresponsive TAb2 tumors were highly enriched with functional tumor-associated macrophages (TAMs), especially M2-TAMs (18). In contrast, sensitive TCh3 tumors contained more CD8 TILs with better effector functions before anti-PD-L1 treatment (18). While anti-PD-L1 did not affect the TME of TAb2 tumors, it significantly increased the number of CD8 TILs in TCh3 tumors (18). These studies suggest that pre-existing immune profiles may dictate the likelihood of a given tumor to respond to anti-PD-L1, consistent with clinical data showing that increased CD8 TILs before ICI treatment correlated with better responses and survival (76).

The obvious question is why these two tumor lines exhibited differential immune profiles before anti-PD-L1 treatment. We performed RNA-seq and whole exome sequencing (WES) and discovered tumor-specific transcriptional, genetic, and epigenetic changes in TAb2 and TCh3 (18). For example, TAb2 tumors expressed a higher level of CSF1, VEGF-C and VEGF-D, and TAb2 tumor cells drastically expanded F4/80^+^ TAMs from bone marrow precursors in a CSF1 and VEGF dependent manner (18). ICI unresponsive TAb2 tumors upregulated distinct signaling pathways that correlate with aggressive tumor phenotypes such as STAT3 pathway (18). However, it remains unknown what tumor-specific changes account for such differential phenotypes, and further studies are warranted. Our studies also suggest that stratifying cancers according to their genetic alterations alone may not be sufficient and evaluating HNSCC tumor-intrinsic cues together with immune profiles in the TME may help better predict ICI responses.

## The effects of host-intrinsic factors on differential ICI responses

Since the adaptive immune system is vastly distinct between different individuals, it is possible that immunological heterogeneity may contribute to the highly variable outcomes of ICI therapy (**Figure 1**). One of the key features of adaptive immunity is “diversity”, generated *via* a somatic DNA recombination process, termed V(D)J recombination. V(D)J recombination occurs in a random stochastic manner in the progenitors of T or B cells, thereby creating a vastly diverse T cell receptor (TCR) or B cell receptor (BCR) repertoire in millions of T or B cells, respectively (77, 78). The TCR of most conventional T cells consists of two different protein chains, an alpha (α) chain and a beta (β) chain, encoded by *TRA* and *TRB*, respectively, and linked by disulfide bonds. A TCR clonotype consists of a unique TCRα and a TCRβ chain with unique V(D)J usage and complementarity-determining region 3 (CDR3). CDR3 encompasses the highly divergent junction of V(D)J recombination and determines TCR specificity; hence, its unique nucleotide or protein sequences can serve as a barcode for individual TCRs.

Studying the formation and diversity of the human TCR repertoire has been difficult due to limited access to human thymus samples and non-feasibility of manipulating variables *in vivo*. Therefore, humanized mouse models were generated by implanting immunodeficient mice with human hematopoietic stem cells (HSCs) and human thymus from the same or different donors to study the development of human T cell repertoire (79). Despite receiving identical HSCs and thymi and having same genetic background and environment, human TCR repertoires were formed in a largely stochastic manner and totally divergent in different recipient mice (79). This means that each individual has an almost completely different TCR repertoire, even in the case of identical twins, an observation which may explain why genetically controlled autoimmune diseases exhibit incomplete penetrance in monozygotic twins (80). Similar to the human TCR repertoires, we predict that individual mice will contain an almost completely different thymic TCR repertoire even if they have identical genetic background (e.g., B6). The initially formed TCR repertoire will be continuously shaped by additional factors including immunization of foreign antigens, pathogen infection, or therapeutic interventions (e.g., CRT). Nonetheless, the peripheral TCR repertoire will remain divergent in individual mice. Prior studies showed that many different TCR clonotypes can react to the same MHC/peptide antigens including model or viral antigens (81, 82). These studies collectively implicate a possibility that different mice could mount anti-tumor immune responses against the same tumor antigens utilizing totally distinct TCR clonotypes. Thus, we propose that the intrinsic differences in diverse TCR repertoires may also contribute to heterogeneous anti-tumor immune responses in different hosts (83). If so, this notion may offer a new explanation for why some hosts would harbor T cells that can eradicate tumors, while others would not.

We proposed “a hole in TCR repertoire” hypothesis to explain the differential ICI responses (83). Currently, there is no data to support the existence of “any hole” in the TCR repertoire given it is dynamic and constantly shaped by various factors. However, there are abundant data supporting immunological differences in individual cancer patients or mouse recipients transplanted with tumors. The frequency of CD8 and CD4 TILs differed in patient samples and positively correlated with clinical outcomes in HNSCCs (23). We also found that the percentage of CD8 TILs varied substantially in HNSCCs with a small fraction containing a high level of CD8 TILs while most patient samples were infiltrated with a low to moderate level of CD8 TILs (20), consistent with an immunosuppressive TME of HNSCCs (19, 84). However, it remains unknown whether the TCR repertoires of CD8 TILs differ in tumor-eradicating vs. tumor-progressing hosts. In this regard, our previous studies showed that Smad4^−/−^ SCCs (A223) elicited divergent responses when transplanted into genetically identical WT B6 mice (20). While a small fraction of tumor-bearing recipients spontaneously rejected the A223 tumor (regressor), most of them underwent tumor progression (progressor) (20). Intriguingly, the top TCR clonotypes were almost mutually exclusive between regressors and progressors (20). Furthermore, both regressor and progressor top TCR clonotypes presented in a recipient-specific manner, suggesting a highly individualized anti-tumor immune response (20). Further studies are warranted to investigate whether the TCR repertoires of CD8 TILs differ in ICI responders vs. non-responders, which may be easier to address using syngeneic mouse models since WT B6 mice have a limited number of MHC class I alleles. The differences in TCR repertoires need to be functionally defined and quantified using an antigen-specific system which can test if distinct TCR clonotypes elicit qualitatively or quantitatively different responses against the same tumor-specific antigen.

It is thus conceivable that immunological heterogeneity (e.g., differences in the TIL TCR repertoire) contributes to the highly variable outcomes of ICI treatment. Why has this idea not been discussed before? Likely because the well-established dogma assumes that there would be sufficient TCR clones that can effectively recognize any tumor-antigen in a given individual due to the enormous size of a TCR repertoire: the estimated T cell number is about 3×10^11^ (85) and the number of TCR clonotypes is about 10^10^ in a given adult (86). Indeed, our adaptive immune system can recognize millions of different pathogens or foreign antigens. However, the effect of immunological heterogeneity is understudied in the context of anti-tumor immunity, and many fundamental questions remain to be addressed, for instance, the actual factors responsible for the highly variable ICI responses remain elusive. Beside TCR differences, BCR may also differ in responders vs. non-responders. Prior studies showed that characteristics of tumor-infiltrating B cells also varied significantly in HNSCCs and correlated with clinical outcomes (87, 88). Overall, future studies are needed to elucidate whether and how host-specific immunological heterogeneity influences differential responses to ICI.

## Discussion

Why are responses to ICI heterogeneous in different cancer patients? What underlying mechanisms lead to such differential responses? These are imperative and fundamental questions for cancer immunology field. Addressing such questions may substantially impact developing new strategies for personalized cancer immunotherapy. We suggest that both tumor-intrinsic and host-intrinsic factors may contribute to differential ICI responses. For instance, by establishing and employing two SCC tumor lines, TAb2 vs. TCh3, both of which harbor *TP53* deletion and *PIK3CA* hyperactivation, we uncovered tumor-intrinsic differences that may underlie the differential responses to ICI (18). However, it still remains to be addressed what tumor-specific genetic or epigenetic changes lead to unresponsiveness in TAb2 or sensitize TCh3 to anti-PD-L1 treatment, and whether such changes are also applicable to the heterogeneous ICI responses in human HNSCCs.

Distinct top TIL TCR clonotypes were found to correlate with tumor eradication vs. tumor progression phenotypes (20). This observation implies that regressor and progressor CD8 TILs might mount drastically different responses by employing distinct TCRs against the same A223 tumor cell line. In line with our observation, prior studies showed that many different TCR clonotypes can react to the same MHC/peptide antigens including model or viral antigens (81, 82). It remains unknown whether the top TCR clonotypes differ in ICI responders vs. non-responders. To address whether the spectrum of TCR clonotypes within regressor or ICI responder provides advantageous recognition of tumor antigens over the spectrum of TCR clonotypes within progressor or ICI non-responder, it would require an antigen-specific model system. Altogether, we propose that stochastic differences in TIL TCR repertoire may be one of several factors that might underlie differential responses to ICI treatment. Of course, this notion does not exclude the contribution of tumor-intrinsic factors, including TMB, tumor immunogenicity, PD-L1 expression or others, to differential ICI responses (21, 50, 89–92); nevertheless, our hypothesis may offer a new perspective to test whether stochastic differences in TCR repertoire contribute to variable ICI responses in different individuals.

## Author contributions

Conceptualization: JW; Writing: JW, ZC, and JJ. All authors contributed to the article and approved the submitted version.

## Funding

This work is supported by UPMC Hillman Cancer Center startup fund to JW, NIH R01-DE027329, R01-DE028420, and R01-DE031947 to JW. ZC was supported by ACS IRG #16-184-56 from the American Cancer Society. The sponsors or funders have no role in the preparation, review, or approval of the manuscript.

## Acknowledgments

We thank Christine Maloney and Dr. Samantha Chen for proofreading the manuscript. We apologize to those whose work was not cited due to length restrictions.

## Conflict of interest

The authors declare that the research was conducted in the absence of any commercial or financial relationships that could be construed as a potential conflict of interest.

## Publisher’s note

All claims expressed in this article are solely those of the authors and do not necessarily represent those of their affiliated organizations, or those of the publisher, the editors and the reviewers. Any product that may be evaluated in this article, or claim that may be made by its manufacturer, is not guaranteed or endorsed by the publisher.
